# Integrating Opioid Use Disorder Treatment Into Primary Care Settings

**DOI:** 10.1001/jamanetworkopen.2023.28627

**Published:** 2023-08-11

**Authors:** Elizabeth J. Austin, Jessica Chen, Elsa S. Briggs, Lori Ferro, Paul Barry, Ashley Heald, Joseph O. Merrill, Geoffrey M. Curran, Andrew J. Saxon, John C. Fortney, Anna D. Ratzliff, Emily C. Williams

**Affiliations:** 1 Department of Health Systems and Population Health, School of Public Health University of Washington, Seattle; 2 Department of Psychiatry and Behavioral Sciences, School of Medicine, University of Washington, Seattle; 3 Advancing Integrated Mental Health Solutions Center, University of Washington, Seattle; 4 Department of Medicine, School of Medicine, University of Washington, Seattle; 5 Departments of Pharmacy Practice and Psychiatry, University of Arkansas for Medical Sciences, Little Rock; 6 Central Arkansas Veterans Health Care System; 7 Center of Excellence in Substance Addiction Treatment and Education, VA Puget Sound, Seattle, Washington; 8 Center of Innovation for Veteran-Centered and Value-Driven Care, Health Services Research and Development, VA Puget Sound, Seattle, Washington

## Abstract

**Importance:**

Medication for opioid use disorder (MOUD) (eg, buprenorphine and naltrexone) can be offered in primary care, but barriers to implementation exist.

**Objective:**

To evaluate an implementation intervention over 2 years to explore experiences and perspectives of multidisciplinary primary care (PC) teams initiating or expanding MOUD.

**Design, Setting, and Participants:**

This survey-based and ethnographic qualitative study was conducted at 12 geographically and structurally diverse primary care clinics that enrolled in a hybrid effectiveness–implementation study from July 2020 to July 2022 and included PC teams (prescribing clinicians, nonprescribing behavioral health care managers, and consulting psychiatrists). Survey data analysis was conducted from February to April 2022.

**Exposure:**

Implementation intervention (external practice facilitation) to integrate OUD treatment alongside existing collaborative care for mental health services.

**Measures:**

Data included (1) quantitative surveys of primary care teams that were analyzed descriptively and triangulated with qualitative results and (2) qualitative field notes from ethnographic observation of clinic implementation meetings analyzed using rapid assessment methods.

**Results:**

Sixty-two primary care team members completed the survey (41 female individuals [66%]; 1 [2%] American Indian or Alaskan Native, 4 [7%] Asian, 5 [8%] Black or African American, 5 [8%] Hispanic or Latino, 1 [2%] Native Hawaiian or Other Pacific Islander, and 46 [4%] White individuals), of whom 37 (60%) were between age 25 and 44 years. An analysis of implementation meetings (n = 362) and survey data identified 4 themes describing multilevel factors associated with PC team provision of MOUD during implementation, with variation in their experience across clinics. Themes characterized challenges with clinical administrative logistics that limited the capacity to provide rapid access to care and patient engagement as well as clinician confidence to discuss aspects of MOUD care with patients. These challenges were associated with conflicting attitudes among PC teams toward expanding MOUD care.

**Conclusions and Relevance:**

The results of this survey and qualitative study of PC team perspectives suggest that PC teams need flexibility in appointment scheduling and the capacity to effectively engage patients with OUD as well as ongoing training to maintain clinician confidence in the face of evolving opioid-related clinical issues. Future work should address structural challenges associated with workload burden and limited schedule flexibility that hinder MOUD expansion in PC settings.

## Key Points

**Question:**

What are multidisciplinary primary care team perspectives on barriers and facilitators to expanding access to medications for opioid use disorder (MOUD)?

**Findings:**

In this survey-based and qualitative study, 4 themes encapsulated multilevel barriers and facilitators associated with primary care team provision of MOUD during implementation: (1) structural barriers delayed or limited primary care team responsiveness to patients needing opioid-related care; (2) patient engagement was more challenging than expected; (3) prescribing clinicians needed ongoing training and tools; and (4) primary care teams had conflicting attitudes about expanding MOUD care.

**Meaning:**

The results of this qualitative study suggest that further support is needed to address the structural barriers to MOUD provision in primary care settings.

## Teaser

This qualitative study explores experiences and perspectives of multidisciplinary primary care teams initiating or expanding medications for opioid use disorder in the US.

## Introduction

Opioid use disorder (OUD) continues to be undertreated despite the availability of effective medications that can be offered in primary care (PC; eg, buprenorphine and naltrexone).^1,2,3^ Primary care offers an access point for OUD identification and treatment. In tandem with other low-barrier and/or specialized treatment facilities, PC can play a role in reducing OUD burden, especially for patients with undetected or untreated OUD.^4,5^ However, adoption of medications for OUD (MOUDs) has been slow in PC settings nationally.^2^

In prior studies, primary care clinicians (PCCs) have reported barriers that stemmed from negative attitudes about prescribing buprenorphine, lack of administrative support, and anticipating an unmanageable influx of patients seeking MOUD.^6,7,8,9,10,11,12,13^ Studies have also repeatedly identified that a lack of access to supportive resources, such as additional clinical team members, is one of the largest barriers to PCC willingness to initiate or expand buprenorphine prescribing.^7,9,10^ However, to our knowledge, few studies have explored perspectives of multidisciplinary PC teams (inclusive of nonprescribing roles) on anticipated and actual barriers experienced throughout MOUD implementation, especially across diverse health settings.^14,15,16,17,18^ Using a cohort of 12 geographically and structurally diverse clinics, we conducted a targeted formative evaluation that explored the implementation experiences of multidisciplinary PC teams that were expanding MOUD services.

## Methods

### Study Overview

Evaluation activities occurred between 2020 and 2022 in a sample of PC clinics that agreed to participating in the Collaborating to Heal Addiction and Mental Health in Primary Care (CHAMP) study. The trial is still ongoing, and primary study results will be reported elsewhere. As part of participation, clinics randomized to the intervention arm (the focus of this article) were asked to initiate or expand MOUD alongside their existing collaborative care services for behavioral health. We then conducted a mixed methods formative evaluation of their MOUD implementation specifically.^19,20^ All activities were approved by the Advarra institutional review board; participants received informed consent materials but per waiver were not required to provide written consent. Study reporting followed recommended guidelines for reporting cohort, survey, and qualitative studies.^21,22,23^

### MOUD Implementation

We used practice facilitation, which is a multifaceted, evidence-based implementation strategy that provides ongoing coaching and technical support to tailor implementation objectives to local context, to support each clinic’s implementation.^24,25^ Each clinic identified a local implementation team and was assigned an external practice facilitator (PF) with prior clinical experience in OUD care delivery who offered tailored implementation support. These PFs had no previous relationship with the clinics. During the preparation phase (approximately 3-6 months), clinics met with the PF twice monthly to develop an implementation plan. The PF, in partnership with clinical mentors, also led a series of 10 training sessions for clinic staff that covered aspects of MOUD delivery. Once clinics launched, they continued monthly implementation meetings and engaged in audit and feedback. The Figure presents a timeline of the study activities.

**Figure.**
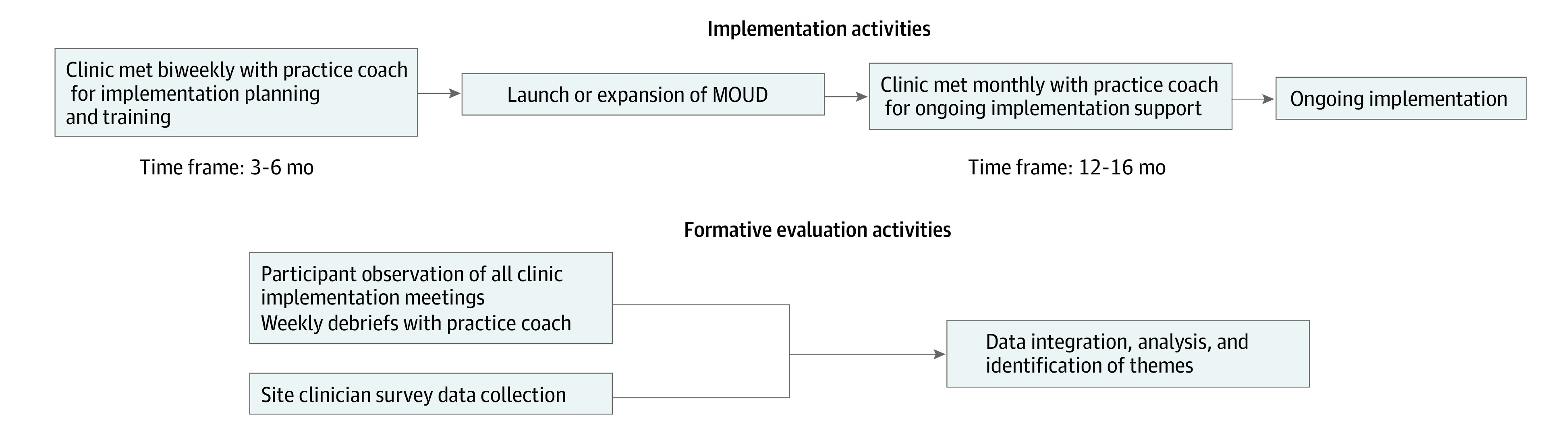
Flow of Study Implementation and Evaluation Activities Each clinic's implementation timeline varied according to clinic goals and readiness. MOUD indicates medications for opioid use disorder.

### Study Design, Data Sources, and Measures

We used a concurrent mixed-methods design in which qualitative and quantitative data were collected in tandem and analyzed separately and then together. Clinics received compensation for participating in the study.

### Qualitative Data

Qualitative data included ethnographic field notes from participant observation of all clinic implementation meetings (n = 179), trainings (n = 97), and internal debriefs with the PF and study team (n = 86). During meetings, trained qualitative researchers (E.J.A., E.S.B., and J.C.) took verbatim notes of all discussions between clinic implementation teams and the PF.

### Quantitative Data

Quantitative data included structured surveys of PC teams that were delivered at the implementation launch. Survey recruitment targeted (1) PCCs (inclusive of prescribing clinicians with MD, DO, PA, and NP licensures), (2) behavioral health care managers (BHCMs; inclusive of clinicians with LICSW, MSW, or PhD licensures who provided therapy and other psychosocial supports but did not prescribe), and (3) consulting psychiatric clinicians (CPs; inclusive of clinicians with MD or NP licensures who provided consultative support to BHCMs and PCCs). All eligible participants were invited to complete the survey electronically. Surveys asked a series of structured questions associated with MOUD care delivery, including items from the Drug Problems Perceptions Questionnaire scale^26^ and items used in the VA’s Stepped Care for Opioid User Disorder Train the Trainer initiative (eTable 1 in Supplement 1).^14,27^

### Data Analysis

Qualitative ethnographic field notes from observations of implementation meetings were analyzed using the rapid assessment process (RAP).^28,29^ Specifically, 2 trained qualitative researchers (E.J.A. and E.S.B.) collected, reviewed, and collaboratively coded field notes using a structured template that was guided by the broad domains of the Consolidated Framework for Implementation Research, which included intervention characteristics, individual characteristics, inner setting, outer setting, and the implementation process.^30^ Data from the field notes were summarized into a site-specific RAP template (eTable 2 in Supplement 1), with representative quotations to support data credibility. Analysis of field notes occurred independently from the primary study analyses to reduce bias. Templates were reviewed weekly using a constant comparison method to monitor anticipated and actual barriers over time. Next, templates were regularly reviewed with the formative evaluation lead (E.C.W.) to identify cross-cutting themes and emergent learnings across RAPs from individual sites. Quantitative survey data were compiled, cleaned, and analyzed descriptively using R (gtsummary package; R Foundation). The team first identified qualitative themes from the ethnographic field notes and completed quantitative analyses separately, then combined the findings to allow for triangulation of data by displaying qualitative themes with parallel quantitative measures to compare perspectives from each data source. Triangulation also worked to reduce social desirability bias inherent to survey measures. Given that the sample of clinics had varying levels of experience with MOUD, we explored potential differences between clinics with and without prior MOUD experience by conducting between-group comparisons for all measures and assessing representation of clinic types across qualitative themes. The final findings were reviewed by the full study team for additional feedback and confirmation. In-text quotes presented for qualitative themes were generated from ethnographic field notes.

## Results

A sample of 62 PC team members completed the survey (response rate, 77%), of whom 21 (50%) had more than 10 years of practice, 41 (66%) identified as female, and 46 (74%) identified as White. Respondents included PCCs (30 [48%]), BHCMs (19 [31%]), and CPs (13 [21%]) from 12 PC clinics located in the eastern (2 [17%]), midwestern (4 [33%]), southern (2 [17%]) and western (4 [33%]) regions of the US (Table 1, Table 2, Table 3, and Table 4; eTable 3 in Supplement 1). Five clinics had no prior experience providing MOUD, whereas the other 7 clinics had prior experience ranging from having a single waivered clinician to having multiple waivered clinicians or formalized care processes (eg, a dedicated MOUD clinic day). Integration of quantitative (survey) and qualitative (ethnographic field note) data identified 4 themes that were associated with PC implementation of MOUD as planned.

**Table 1.** Clinic Characteristics

Clinic characteristics	No. (%) (n = 12)^a^
Geographic region	
East	2 (16.7)
Midwest	4 (33.3)
South	2 (16.7)
West	4 (33.3)
Clinic ownership	
Local, county, or community government	1 (8.3)
Private, for-profit hospital/hospital system	1 (8.3)
Not-for-profit organization/foundation	10 (83.3)
Special designation or accreditation	
Federally qualified health center	3 (25.0)
Patient-centered medical home	4 (33.3)
Other^b^	3 (25.0)
MOUD experience before study launch?	
Yes	7 (58.3)
No	5 (41.7)
Average patient census	
<5000	2 (16.7)
5000-10 000	5 (41.7)
>10 000	5 (41.7)
Residents and/or trainees?	5 (41.7)
Academic medical center–affiliated?	
Yes	3 (25.0)

Abbreviation: MOUD, medication for opioid use disorder.

^a^ Percentages are calculated from respective characteristic total counts, so they may not summate to 100.

^b^ Other check box responses included: part of an accountable care organization and previously a primary care medical home. One response was left blank.

**Table 2.** Survey Participant Characteristics

Participant characteristics^a^	No. (%) (n = 62)^b^
Role at clinic	
Psychiatric consultant	13 (21.0)
Behavioral health care manager	19 (30.7)
Primary care clinician	30 (48.4)
Years of practice	
<5	8 (13.0)
5-10	23 (37.1)
11-20	21 (33.9)
>20	10 (16.1)
Age, y	
25-34	9 (14.5)
35-44	28 (45.2)
45-54	14 (22.6)
>55	11 (17.8)
Race and ethnicity	
American Indian or Alaskan Native	1 (1.6)
Asian	4 (6.5)
Black or African American	5 (8.1)
Hispanic or Latino	5 (8.1)
Native Hawaiian or Other Pacific Islander	1 (1.6)
White	46 (74.2)
Gender identity	
Female	41 (66.1)
Male	21 (34.0)

^a^ All responses were self-reported.

^b^ Percentages are calculated from respective characteristic total counts and rounded, so they may not summate to 100.

**Table 3.** Survey Results: Primary Care Team–Reported Attitudes Toward OUD Care Delivery

Attitudes toward working with patients with OUD (DPPQ)^a^	No. (%)					
	Strongly disagree	Disagree	Slightly disagree	Slightly agree	Agree	Strongly agree
Domain 1: role adequacy						
I believe I have a working knowledge of opioids and opioid-related problems	0	2 (3)	2 (3)	12 (20)	29 (48)	15 (25)
I believe I know how to counsel opioid users over the long-term	0	5 (8)	8 (13)	17 (28)	20 (33)	10 (17)
Domain 2: role support						
If I felt the need when working with opioid users, I could easily find someone who would be able to help me formulate the best approach for an opioid user	0	4 (6)	2 (3)	13 (22)	28 (47)	13 (22)
Domain 3: job satisfaction						
In general, I have less respect for opioid users than for most other patients I work with	38 (63)	19 (32)	1 (2)	2 (3)	0	0
In general, one can get satisfaction from working with opioid users	0	2 (3)	0	8 (13)	29 (48)	21 (35)
Domain 4: role related self esteem						
I often feel uncomfortable when working with opioid users	14 (23)	21 (35)	6 (10)	16 (27)	3 (5)	0
Domain 5: role legitimacy						
I feel I have the right to ask patients questions about their opioid use when necessary	0	0	1 (2)	6 (10)	29 (48)	24 (40)

Abbreviations: DPPQ, the Drugs Problems Perception Questionnaire; OUD, opioid use disorder.

^a^ Two observations were missing.

**Table 4.** Survey Results: Primary Care Team–Reported Beliefs Toward OUD Care Delivery

Beliefs about providing MOUD	No. (%)			
	Strongly disagree	Disagree	Agree	Strongly agree
Delivering medications to treat OUD in my clinic:				
Is important	0	2 (3)	21 (34)	39 (63)
Will save lives	0	1 (2)	16 (26)	45 (73)
Is time consuming	2 (3)	20 (32)	32 (52)	8 (13)
Detracts from my clinical responsibilities	20 (33)	35 (56)	7 (11)	0
Is more dangerous and/or uncomfortable than management of other chronic diseases	18 (29)	36 (58)	8 (13)	0
Can be done successfully in primary care	0	0	31 (50)	31 (50)
Without onsite formal drug counseling, office-based buprenorphine treatment is ineffective	18 (29)	28 (45)	12 (19)	4 (7)
Abstinence from using opioids (including buprenorphine) is the principal goal of treatment for OUD	25 (40)	23 (37)	10 (16)	4 (7)
I am concerned about being able to accommodate patients seeking OUD treatment at my clinic for the following reasons (select all that apply):				
Waivered prescriber will not have the DEA waiver capacity to meet demand	NA	5 (8)	NA	NA
Clinicians will not have the caseload to accommodate patients seeking OUD care	NA	20 (32)	NA	NA
Clinic will experience an influx of new patients seeking OUD care	NA	18 (29)	NA	NA

Abbreviations: DEA, US Drug Enforcement Agency; MOUD, medication for opioid use disorder; NA, not applicable; OUD, opioid use disorder.

### Theme 1: Limitation of PC Team Ability to Offer Patients Rapid Access to OUD Care Within Existing Administrative Structures

Primary care teams identified multiple ways the administrative structures in their settings limited their responsiveness to patients with OUD. Many PC teams described a limited capacity to absorb new patients. As 1 clinician said, “my practice has been really busy right now… it’s been tough to find openings for my current patients as it is” (PCC, site 6). Other clinicians described challenges with closed panels or limited panel sizes, often established by their health systems. In survey responses, 20 clinicians (32%) expressed concerns that the potential volume of patients seeking OUD treatment would be beyond what their clinic could accommodate.

Primary care teams also found that inflexible appointment scheduling processes, which were generally created for brief annual or problem-focused visits, created challenges. For example, a patient who is new to the clinic may need to complete an arduous intake process before gaining access to available appointments, which may deter patients seeking MOUD. As 1 clinician said, “it’s not easy to get an appointment as a new patient” (PCC, site 1). Additionally, teams described that their schedules were often full, preventing them from quickly accommodating patients who were ready to initiate treatment with MOUD, leading some patients to seek care elsewhere. In other examples, clinicians described the urgency to ensure that patients experiencing withdrawal from opioids were seen quickly to reduce discomfort and avoid patients falling out of care, yet their current schedules did not allow for urgent visits. One PCC characterized this, saying, “It gets a little bit cumbersome when your schedule is blocked… open access would be great; it’s just getting the hospital to buy in… that would be nice to have a little bit of breathing space there instead of putting everyone else behind, especially when they’re withdrawing in the next room” (PCC, site 2).

Care teams described that increasing their schedule flexibility was at odds with health system goals for productivity, including pressure to have schedules fully booked with appointments. As 1 BHCM described, “the idea that they will leave an hour and a half open in my schedule every day or every other day knowing they may not be filled, that’s not cost effective” (BHCM, site 5). Some clinicians acknowledged that scheduling inflexibility hindered their ability to engage patients, saying: “you really have to strike when the iron’s hot and maybe we’re losing patients that way” (CP, site 3).

### Theme 2: Challenges With Patient Engagement in OUD Treatment

As PC teams launched or expanded their provision of MOUD, they identified fewer patients with OUD than anticipated. One clinician summarized that in terms of OUD patient volume, “we’re not seeing it” (CP, site 12). Some clinicians believed that patients may not feel comfortable seeking MOUD in primary care, partially due to stigma from clinic-level efforts to reduce opioid prescribing. As 1 described, “[city name] is pretty small, everybody knows that I will not give chronic opioids, they will even say they don’t want to see me because they know I won’t do that” (PCC, site 2). When patients with OUD were identified, clinic teams struggled to engage them in MOUD. As 1 BHCM described, “we can’t just find these people for months and months. […] I’m spending 3 weeks, 4 weeks, trying to get them in” (BHCM, site 10). Another shared, “it’s frustrating when patients don’t show up when they have been referred” (BHCM, site 4).

Clinicians expressed the need to increase awareness about the availability of MOUD in PC and reduce community-level stigma that has historically deterred patients. One said, “I think there’s still a lot of lacking awareness in general in our area and even in the ERs and from all providers” (PCC, site 8). Another acknowledged that more effort is needed to help clinic staff approach patients with OUD in less stigmatizing ways, saying, “I think we’ve got a little bit of a ways to go with just stigma and educating primary care clinics in general” (behavioral health director, Site 11).

### Theme 3: Training and Support for Prescribing Clinicians on MOUD Conversations With Patients

Many PC teams expressed low confidence for identifying and discussing OUD treatment. Care teams acknowledged that some clinicians “don’t have experience or feel comfortable, or maybe don’t understand use disorder criteria” (PCC, site 8). Another PCC shared, in reference to discussing a potential OUD diagnosis with patients, that “it’s a struggle for us to feel empowered to have those conversations since we’re so new” (PCC, site 2). Survey data also reflected clinician hesitancy, with only half of clinicians expressing confidence in their ability to counsel patients long term and 19 (32%) expressing discomfort when interacting with patients with OUD. Clinicians at clinics without prior MOUD experience were more likely to report low knowledge (20% vs 0%; *P* < .001), greater discomfort (60% vs 18%; *P* < .02), and limited confidence when counseling patients with OUD (35% vs 15%; *P* < .001). Yet even clinicians with greater MOUD experience felt challenged by some clinical MOUD scenarios they encountered with patients. As 1 PCC said, “I swear nobody’s simple, like can’t someone just have straightforward depression and [buprenorphine] issues?” (PCC, site 4).

Clinicians described many scenarios in which they wanted more guidance on how to talk about OUD with patients. For example, while 48 (77%) did not believe abstinence was the primary goal of OUD care, some clinicians felt unsure of how to navigate potential diversion (ie, patients sharing or selling their OUD medications to others). One PCC said, “I’m afraid to ask if [the patient is] selling it. I’m scared to breach the subject of ‘well what is happening to the [buprenorphine].’ I don’t want to be so judgy, I want to be open” (PCC, site 8). Primary care teams highlighted that variation in the source of opioid use (ie, heroin vs prescription opioids) created complexity in communicating about MOUD. One PCC described “I have my patients that have been using street drugs where I don’t have to explain any of that, vs my patients that are on prescribed opioids, I have to use visuals, I draw the little receptors …explaining these ideas of tolerance and withdrawal are not terms that my [patients with chronic pain] are as familiar with or comfortable with discussing” (PCC, site 4).

Yet most clinicians reported that they would be able to identify supports in their clinic, such as internal or external mentors. As 1 shared, “I wouldn’t have had the guts to do it, honestly, without a mentor” (PCC, site 2). Primary care teams also identified that electronic medical record templates for OUD, such as templates that describe the *Diagnostic and Statistical Manual of Mental Disorders* (Fifth Edition) criteria for OUD diagnosis, can help with clinician confidence. Additionally, care teams highlighted the need for more patient education tools to help with treatment discussions. As 1 PCC suggested, “I think it’d be great to have a handout on ‘why I should use [buprenorphine]’” (PCC, site 8).

### Theme 4: Variable Attitudes Within PC Teams Toward Expanding MOUD in Their Practice

When surveyed, PC teams unanimously agreed that MOUD can be successfully delivered in PC, and 60 (97%) expressed that MOUD was important for PC settings. All clinicians also agreed that they were able to help patients with OUD, and 57 (95%) perceived OUD care to be rewarding to provide.

However, in practice, PC teams had conflicting perspectives on how MOUD integrated into their already busy workloads. More than 65% of clinicians perceived providing opioid-related care to be time consuming and felt further challenged to take on more patients while facing barriers, such as scheduling inflexibility and limited administrative supports. One PCC said, “to be honest I got a little bit of the ‘oh god something else to do’” (PCC, site 12). Another described the stress clinicians felt when expanding OUD care, saying, “I don’t know if I would say resistance but a general overwhelm and fitting in what they need to do in their visit with the patient … the time restrictions are stressful” (CP, site 3).

Clinicians responded to these conflicting attitudes in different ways. For some, it increased their resistance to expanding MOUD without other forms of workload relief. As 1 PCC described, “Our faculty group as a whole has expressed that that’s not the direction they want for our clinic, we already provide more psychiatric care and addiction medicine than other clinics, but we can’t be like the addiction medicine clinic in town either” (PCC, site 9).

For others, the importance of expanding MOUD remained paramount, despite the barriers they faced. One clinician shared this attitude, saying “it’s just going to make life interesting, and we’re just going to make it work” (PCC, site 4).

## Discussion

In a cross-national implementation study in 12 PC clinics, we used mixed methods to characterize multidisciplinary PC team experiences in integrating MOUD into primary care. Consistent with prior literature, we identified multiple factors that were associated with the expansion of MOUD care, including clinician confidence, patient engagement, and barriers to scheduling and capacity. We also found that most clinicians believed providing MOUD in PC settings was important and should not include abstinence as the primary goal of treatment. Compared with prior studies, these findings represent a shift toward less stigmatizing views on OUD care among PC teams.^7,8^ While clinicians also expressed concerns about their capacity to absorb new patients, including those with OUD, most struggled to identify patients eligible for treatment, and some clinicians felt discouraged by the low yield. Documenting these counterfactual experiences, such as the anticipated vs actual volume of patients seeking OUD care, may help to change the narrative around the feasibility of providing MOUD in PC.^7,9,31,32,33^

This study’s findings also suggest a need to better address barriers that lead PC teams to feel burdened by MOUD. Although all of the clinicians believed that MOUD could be successfully delivered in PC settings, many also held negative attitudes about how MOUD increased their workload without other forms of workload relief. In a US Department of Veteran Affairs–based study, 37.5% of PC team members perceived MOUD to be time consuming; this concern was almost double (65%) within the present study’s sample.^14^ Attitudes associated with self-efficacy (ie, beliefs about the capacity to successfully deliver OUD care), role identity, and workload are intertwined and can be associated with decreased clinician motivation over time, especially when reinforced by unsupportive clinic environments and persistent stigma.^34^ Expanding MOUD in PC will likely require thoughtful strategies at the system level, such as higher rates of reimbursement for MOUD provision that compensate for additional efforts needed to support patient engagement.^33,35,36,37,38^ For example, variation in Medicaid reimbursement has substantially contributed toward the adoption of integrated care practices for behavioral health, a pattern potentially mirrored for OUD care.^39^ Finally, future work should continue to unpack the associations between perceived burden and stigma, including stigma enacted at the institutional level. While this study’s data showed less stigmatizing attitudes among clinicians compared with prior work, stigma at the institutional and community levels will continue to stymie MOUD implementation efforts for patients and PC teams if not addressed.

### Limitations

There were several limitations in this study. Qualitative data are intended to characterize perspectives but not to be generalizable. Pairing qualitative and quantitative data increases the confirmability of findings; however, the findings may not be applicable to all settings. Additionally, this study reflects an analysis of a formative evaluation in an ongoing implementation; further research by this team and others should also evaluate metrics that characterize receipt of MOUD and care engagement across diverse PC settings and explore the association of local context with implementation outcomes.

## Conclusions

This qualitative study identified multiple factors that are associated with and at times impede MOUD implementation in PC settings. Future work should consider opportunities at the organizational and system levels to increase incentives and flexibility for PC teams looking to adopt or expand opioid-related care.

## Notes

Supplement 1. **eTable 1.** Summary of measures included in provider survey **eTable 2.** Example Rapid Assessment Process (RAP) template for qualitative coding **eTable 3.** Full Drug Problems Perceptions Questionnaire (DPPQ) results

Supplement 2. Data sharing statement
