# Supplementary material for: Integrating Opioid Use Disorder Treatment Into Primary Care Settings
Source: JAMA Netw Open. 2023 Aug 11;6(8):e2328627. doi: 10.1001/jamanetworkopen.2023.28627 (PMC10422185; doi:10.1001/jamanetworkopen.2023.28627)
Supplement: Supplement 1. — eTable 1. Summary of measures included in provider survey eTable 2. Example Rapid Assessment Process (RAP) template for qualitative coding eTable 3. Full Drug Problems Perceptions Questionnaire (DPPQ) results [file jamanetwopen-e2328627-s001.pdf]

Supplemental Online Content

Austin EJ, Chen J, Briggs ES, et al. Integrating opioid use disorder treatment into primary care settings. *JAMA Netw Open*. 2023;6(8):e2328627. doi:10.1001/jamanetworkopen.2023.28627

eTable 1. Summary of measures included in provider survey

eTable 2. Example Rapid Assessment Process (RAP) template for qualitative coding

eTable 3. Full Drug Problems Perceptions Questionnaire (DPPQ) results

This supplemental material has been provided by the authors to give readers additional information about their work.

© 2023 Austin EJ et al. *JAMA Network Open.*

eTable 1. ***Summary of measures included in provider survey***

Measure	Description	Example items	Goal for use
**Drug Problems Perceptions Questionnaire** (DPPQ) (Watson, 2007)	A psychometrically validated 20-item scale that assesses provider attitudes about OUD care delivery across the domains of 1) *role adequacy* (i.e., the extent to which providers feel adequately prepared for their role, including having appropriate knowledge and skills), 2) *role legitimacy* (i.e., the extent to which providers perceive work as being an appropriate part of their job), 3) *job satisfaction* (i.e., the extent to which providers enjoy their work), 4) *role support* (i.e. the extent to which providers feel supported by their colleagues), and 5) *role-related self-esteem* (i.e., the extent to which providers have confidence in their care delivery skills)	*"I feel I know how to counsel opioid users over the long-term"*<br><br>*"I often feel uncomfortable when working with opioid users."*	The full DPPQ scale was included to assess all domains of provider attitudes towards their role delivering OUD care.
**VA Stepped Care for Opioid Use Disorder Train the Trainer** (SCOUTT) (Gordon, 2020)	Researchers in the VA health system developed a series of structured items to assess provider attitudes and beliefs towards the practice of delivering MOUD, including a focus on medications for OUD that can be delivered in primary care settings.	*"Delivering medications to treat OUD is important"*<br><br>*"Delivering medications to treat OUD is time consuming"*	Selected items from SCOUTT were included to assess provider perspectives on MOUD delivery and fit with practice.

© 2023 Austin EJ et al. *JAMA Network Open.*

eTable 2. ***Example Rapid Assessment Process (RAP) template for qualitative coding***

**RAP Details**	
Site name:	[full site name]
Site roles involved:	[list all site roles involved in implementation meetings]
Dates of review:	[date range of observation data included in RAP]
**Summary of Observation Data**	
Intervention characteristics	[summary points and exemplar quotes]
Individual characteristics	[summary points and exemplar quotes]
Inner Setting	[summary points and exemplar quotes]
Outer Setting	[summary points and exemplar quotes]
Implementation process	[summary points and exemplar quotes]
**Analyst Notes & Questions**	
[memo of data interpretation and other relevant notes]	

© 2023 Austin EJ et al. *JAMA Network Open.*

eTable 3. ***Full Drug Problems Perceptions Questionnaire (DPPQ) Results***

Attitudes towards working with patients with OUD (DPPQ)^*	Strongly Disagree No. (%)	Disagree No. (%)	Slightly Disagree No. (%)	Slightly Agree No. (%)	Agree No. (%)	Strongly Agree No. (%)
**Domain 1: Role Adequacy**						
I feel I have a working knowledge of opioids and opioid – related problems.	0	2 (3%)	2 (3%)	12 (20%)	29 (48%)	15 (25%)
**I feel I know enough about…**						
The causes of drug problems to carry out my role when working with opioid users	1 (2%)	1 (2%)	0	12 (20%)	31 (52%)	15 (25%)
The physical effects of drug use to carry out my role when working with opioid users	0	1 (2%)	2 (3%)	15 (25%)	31 (52%)	11 (18%)
The psychological effects of drug use to carry out my role when working with opioid users	0	1 (2%)	2 (3%)	10 (16%)	33 (53%)	14 (23%)
The factors which put people at risk of developing drug problems to carry out my role when working with opioid users	0	1 (2%)	0	12 (20%)	35 (58%)	12 (20%)
I feel I know how to counsel opioid users over the long-term	0	5 (8%)	8 (13%)	17 (28%)	20 (33%)	10 (17%)
I feel I can appropriately advise my patients about opioids and their effects.	0	0	2 (3%)	14 (23%)	31 (52%)	13 (22%)
**Domain 2: Role Support**						
**If I felt the need when working with opioid users, I could easily find someone…**						
With whom I could discuss any personal difficulties that I might encounter	0	4 (7%)	6 (10%)	6 (10%)	27 (45%)	17 (28%)
Who would help me clarify my professional responsibilities	0	3 (5%)	4 (7%)	11 (18%)	29 (48%)	13 (22%)
Who would be able to help me formulate the best approach for an opioid user	0	4 (7%)	2 (3%)	13 (22%)	28 (47%)	13 (22%)
**Domain 3: Job Satisfaction**						
I feel I am able to work with opioid users as well as other patient groups	0	0	0	16 (27%)	24 (40%)	20 (33%)
**In general…**						
I have less respect for opioid users than for most other patients I work with.	38 (63%)	19 (32%)	1 (2%)	2 (3%)	0	0
One can get satisfaction from working with opioid users.	0	2 (3%)	0	8 (13%)	29 (48%)	21 (35%)
It is rewarding to work with opioid users.	0	0	3 (5%)	16 (27%)	27 (45%)	14 (23%)
I feel I can understand opioid users.	0	1 (2%)	4 (7%)	18 (31%)	24 (41%)	12 (20%)
**Domain 4: Role Related Self Esteem**						
I feel that there is little I can do to help opioid users	26 (43%)	27 (45%)	6 (10%)	0	0	1 (2%)
I often feel uncomfortable when working with opioid users.	14 (23%)	21 (35%)	6 (10%)	16 (27%)	3 (5%)	0
All in all, I am inclined to feel I am a failure with opioid users.	29 (48%)	22 (37%)	5 (8%)	3 (5%)	0	1 (2%)
**Domain 5: Role Legitimacy**						
**I feel I have the right…**						
To ask patients questions about their opioid use when necessary	0	0	1 (2%)	6 (10%)	29 (48%)	24 (40%)
To ask patients for any information that is relevant to their opioid related problems	0	0	1 (2%)	8 (13%)	28 (47%)	23 (38%)

^DPPQ refers to the Drugs Problems Perception Questionnaire
**2 observations missing*

© 2023 Austin EJ et al. *JAMA Network Open.*
