# Supplementary material for: Integrating Opioid Use Disorder Treatment Into Primary Care Settings
Source: JAMA Netw Open. 2023 Aug 11;6(8):e2328627. doi: 10.1001/jamanetworkopen.2023.28627 (PMC10422185; doi:10.1001/jamanetworkopen.2023.28627)
Supplement: Supplement 2. — Data sharing statement [file jamanetwopen-e2328627-s002.pdf]

Data Sharing Statement

Austin. Integrating Opioid Use Disorder Treatment Into Primary Care Settings. *JAMA Netw Open*. Published August 11, 2023. doi:10.1001/jamanetworkopen.2023.28627

Data
Data available: No

Additional Information
Explanation for why data not available: We are willing to curate datasets based on individual requests.
